# Icaritin Induces Anti-tumor Immune Responses in Hepatocellular Carcinoma by Inhibiting Splenic Myeloid-Derived Suppressor Cell Generation

**DOI:** 10.3389/fimmu.2021.609295

**Published:** 2021-02-26

**Authors:** Huimin Tao, Mingyu Liu, Yuan Wang, Shufeng Luo, Yongquan Xu, Bin Ye, Limin Zheng, Kun Meng, Lian Li

**Affiliations:** ^1^Ministry of Education Key Laboratory of Gene Function and Regulation, School of Life Sciences, Sun Yat-sen University, Guangzhou, China; ^2^School of Medicine, Guangzhou First People's Hospital, South China University of Technology, Guangzhou, China; ^3^State Key Laboratory of Oncology in South China, Collaborative Innovation Center for Cancer Medicine, Sun Yat-sen University Cancer Center, Guangzhou, China; ^4^Beijing Shenogen Biomedical Ltd, Beijing, China

**Keywords:** icaritin, hepatocellular carcinoma, extramedullary hematopoiesis, MDSC, immunotherapy

## Abstract

Recent studies have demonstrated that splenic extramedullary hematopoiesis (EMH) is an important mechanism for the accumulation of myeloid-derived suppressor cells (MDSCs) in tumor tissues, and thus contributes to disease progression. Icaritin, a prenylflavonoid derivative from plants of the *Epimedium* genus, has been implicated as a novel immune-modulator that could prolong the survival of hepatocellular carcinoma (HCC) patients. However, it is unclear whether icaritin achieves its anti-tumor effects via the regulation of MDSCs generated by EMH in HCC. Here, we investigated the anti-tumor potential of icaritin and its mechanism of action in murine HCC. Icaritin suppressed tumor progression and significantly prolonged the survival of mice-bearing orthotopic and subcutaneous HCC tumors. Rather than exerting direct cytotoxic activity against tumor cells, icaritin significantly reduced the accumulation and activation of tumoral and splenic MDSCs, and increased the number and activity of cytotoxic T cells. Mechanistically, icaritin downregulates the tumor-associated splenic EMH, thereby reducing the generation and activation of MDSCs. The inhibitory effects of icaritin on human MDSCs *in vitro* were verified in short-term culture with cord-blood derived hematopoietic precursors. Furthermore, icaritin synergistically enhanced the therapeutic efficacy of immune checkpoint blockade therapy in HCC mice. These findings revealed that icaritin dampens tumoral immunosuppression to elicit anti-tumor immune responses by preventing MDSC generation via the attenuation of EMH. Thus, icaritin may serve as a novel adjuvant or even a stand-alone therapeutic agent for the effective treatment of HCC.

## Introduction

Hepatocellular carcinoma (HCC) is among the most common types of neoplasm worldwide, with increasing incidence and extremely poor prognosis (1, 2). As an inflammation-associated cancer, it has been well-evidenced that the immune microenvironment of HCC tissue plays a key role in disease progression and efficacy of clinical treatments (3). Recently, immunotherapy targeting the tumor microenvironment has revealed new opportunities for therapeutics, and has achieved promising clinical responses against HCC (4, 5). However, only a small fraction of patients with HCC have benefited from these treatments (6), highlighting the urgent requirement for the identification of novel immunotherapeutic targets with therapeutic efficacy so as to improve the clinical outcome of patients with HCC.

Myeloid cells are a group of heterogeneous immune cells that have been characterized as crucial regulators of cancer immune responses (7–9). These cells promote cancer cell stemness (10), facilitate angiogenesis and metastasis (9, 11–13), and impact virtually all types of cancer therapy (14–17). These cells are generally short-lived and must be continuously replenished throughout cancer progression (18). Therefore, cancer is associated with a profound myeloid response resulting in the expansion of tumor-associated myeloid cells that promote disease progression (19, 20). We have recently observed that circulating hematopoietic precursors exhibit myeloid bias, and patients with solid tumors exhibit a skew toward myeloid differentiation (19). Furthermore, splenic extramedullary hematopoiesis (EMH) accommodates myeloid-biased hematopoiesis, which facilitates the generation of functional myeloid-derived suppressor cells (MDSCs), and plays a critical role in disease progression (21). Due to this effect of EMH on the immune status of the tumor microenvironment (20, 22), the abrogation of EMH may have potential to be implemented therapeutically.

Icaritin is a hydrolytic product of icariin isolated from plants of the *Epimedium* genus, and has been used traditionally in Chinese herbal medicine. Previous studies have shown that icaritin possesses a wide range of therapeutic capabilities, including the promotion of bone repair and anti-inflammatory effects (23). Icaritin exhibits direct cytotoxic activity against certain types of cancer cells (23–26). However, recent research suggests that icaritin may affect the activity of a variety of immune cell types (27, 28) and serve as a novel immune-modulator in HCC (29). Indeed, the results of recent clinical trials of icaritin in patients with advanced HCC (NCT02496949) showed that patients who demonstrated prolonged survival also exhibited decreased neutrophil and increased lymphocyte counts after treatment (30). This suggests that icaritin may possess an immunomodulatory activity. Icaritin was recently shown to reduce the frequency of tumoral MDSCs that resulted in reduced tumor progression in murine melanoma (28); however, the underlying mechanism remains largely unknown.

Given the lack of knowledge on this subject, we investigated the anti-tumor potential of icaritin in murine HCC. Here, we demonstrate that icaritin inhibits tumor growth and prolongs the survival of mice with HCC. Icaritin elicits anti-tumor immune responses by inhibiting the infiltration and immunosuppressive activity of MDSCs; thus, icaritin synergistically enhances the efficacy of immune checkpoint blockade therapy. Furthermore, we provide evidence that icaritin attenuates splenic EMH to decrease the generation of MDSCs in tumor-bearing mice. The inhibitory effects of icaritin on the generation of human MDSCs were verified in a short-term culture model using cord-blood derived CD34^+^ cells.

## Materials and Methods

### Cell Lines and Cell Culture

The murine HCC cell line, Hepa1–6, was originally obtained from the American Type Culture Collection (Virginia, USA). The murine ascites hepatoma cell line H22 was purchased from the Cell Bank of Type Culture Collection (Chinese Academy of Sciences, Shanghai, China). Hepa1–6 cells were cultured in Dulbecco's modified Eagle's medium (DMEM, Life Technologies, C11995500BT) supplemented with 10% fetal bovine serum and 100 μg/mL penicillin/streptomycin (Hyclone) at 37°C in a humidified atmosphere of 5% CO_2_. H22 cells were amplified by intraperitoneal transplantation of 1 × 10^5^ tumor cells into BALB/c mice to trigger ascetic growth.

### Generation of Human Cord Blood-derived MDSCs

The cord blood CD34^+^ cells were expanded as described previously (19). In brief, human CD34^+^ cells were isolated from the cord blood mononuclear cells using a direct CD34 progenitor cell isolation kit (Miltenyi Biotec), and cultured in hematopoietic stem cell expansion media (StemSpan SFEM; Stem Cell Technologies) supplemented with 100 ng/mL stem cell factor, 100 ng/mL Fms-like tyrosine kinase 3, 100 ng/mL thrombopoietin, and 20 ng/mL IL-3 (R&D Systems) for 9 days (31). After expansion, CD34^+^ cells were plated at 2.5 × 10^5^ cells/well in 24-well plates in DMEM with 2.5 μM icaritin for 2 h, and cultured with GM-CSF (40 ng/mL) and IL-6 (40 ng/mL) at 37°C in 5% CO_2_ for 3 days.

### Mice

Female C57BL/6 (B6) and BALB/c mice (6–8 weeks of age) were purchased from Guangdong Medical Laboratory Animal Center (Guangzhou, China). All mice were maintained in specific pathogen-free conditions in the animal facilities of Sun Yat-sen University Cancer Center (Guangzhou, China).

### Tumor Challenge and Treatments

The orthotopic tumor model was established via a subcapsular intrahepatic injection of 5 × 10^5^ Hepa1–6 cells, suspended in 25 μL of 50% Basement Membrane Extract (Trevigen), into the left lobe of the liver of anesthetized 6–8 week old B6 mice. For the subcutaneous tumor model, 1 × 10^6^ Hepa1–6 or 2 × 10^5^ H22 cells suspended in 100 μL phosphate buffered saline (PBS) were injected subcutaneously into the right flank of B6 mice or BALB/c mice. A total of 5 days after tumor cell transplantation, when tumors were palpable or subcutaneous tumors reached 100 mm^3^, the mice were randomly divided into treatment groups. Icaritin (ICT; Beijing Shenogen Biomedical Ltd, China) treatment, was administered daily at 70 mg/kg by gavage for up to 3 weeks. Corn oil was used as the vehicle control. Anti-PD-1 antibody (clone RMP1-14; BioXCell) was administered at 10 mg/kg by intraperitoneal injection a total of three times at 3 day intervals. Once tumors were palpable, tumor growth was monitored every other day for 17 days using calipers. The survival time of tumor-bearing mice was recorded from the day of inoculation. The mice were sacrificed if the tumor diameter exceeded 1.5 cm or if the mice showed any signs of pain. Tumor volumes were calculated using the following formula: Volume = (length × width^2^)/2.

### Flow Cytometry and Cell Isolation

Flow cytometry was performed as previously described (21). Details of the antibodies used for flow cytometry are listed in Supplementary Table 1. Cells from the tumor or spleen tissue, or *in vitro* cultured cells were homogenized and filtered to create a single cell suspension followed by resuspension in 100 μL PBS (supplemented with 1% heat-inactivated FBS and 2 mM EDTA) for antibody staining. For intracytoplasmic IFN-γ detection, cells were stimulated in RPMI-1640 supplemented with 10% FBS and 0.5% Leukocyte Activation Cocktail at 37°C for 4.5 h. After *in vitro* stimulation, the cells were stained with surface marker antibodies followed by fixation and permeabilization using the Fixation/Permeabilization Solution Kit (BD Cytofix/Cytoperm) prior to staining with the IFN-γ antibody. All data were acquired using a Cytoflex S flow cytometer (Beckman Coulter) and analyzed using the FlowJo Software (Tree Star).

To sort polymorphonuclear MDSCs (PMN-MDSCs), Gr-1^+^ cells were first sorted using the anti-Gr-1-coupled MACS beads (Miltenyi Biotec), and then stained with anti-CD11b and anti-Ly6G antibodies for sorting by a MoFlo XDP flow cytometer (Beckman Coulter). The purity of the sorted PMN-MDSC population was evaluated by flow cytometry and exceeded 95%.

### Coculture of PMN-MDSCs With T Cells

Naïve splenocytes were labeled with 2.5 μM CFSE (Invitrogen Molecular Probes) and cultured in RPMI-1640 supplemented with 10% FBS, 20 U/mL recombinant IL-2 (Thermo Fisher Scientific), 1 μg/mL anti-CD3 (Thermo Fisher Scientific), and 5 μg/mL anti-CD28 (Thermo Fisher Scientific). Splenocytes were cultured alone or with freshly sorted PMN-MDSCs at the indicated ratio for 84 h at 37°C in a 5% CO_2_ humidified atmosphere. Subsequently, CFSE dilution was assessed using a Cytoflex S flow cytometer (Beckman Coulter); splenocyte number was determined using the FlowJo Software (version 13; Tree Star).

### Western Blotting

The immunoblotting technique was performed as previously described (32). Equal amounts of cellular protein were separated by SDS-PAGE on a 10% gel, and immunoblotted with the following antibodies: β-actin (AC-15; Boster Biological Technology), t-STAT3 (124H6; Cell Signaling Technology), p-STAT3 (D3A7; Cell Signaling Technology), and Arg-1 (D4E3M; Cell Signaling Technology). Immunoblots were visualized using an ECL kit (Thermo Fisher Scientific). Western blotting results were quantified using the Image J software (NIH, Bethesda, MD, USA).

### Statistical Analysis

Data were analyzed using GraphPad Prism (version 8). All values are expressed as the mean ± SEM. All statistical tests were performed as two sided. For data that were normally distributed, we applied the Student's *t*-test; the non-parametric exact Wilcoxon signed-rank test was used to compare data not normally distributed. For multiple comparisons (including multiple two-group comparisons shown in the same panel), a one-way or two-way ANOVA (for parametric data) was followed by the Bonferroni's correction (only two groups were compared) or the Dunnett's test (all groups were compared to one control group). Cumulative survival time was estimated by the Kaplan-Meier method, and the log-rank test was applied to compare the groups. *P* < 0.05 were considered statistically significant.

### Ethics Approval and Consent to Participate

All animal experiments were performed according to state guidelines and approved by the IACUC of the Sun Yat-sen University Cancer Center. Human cord blood was obtained from the First Affiliated Hospital of Sun Yat-Sen University. All samples were coded anonymously under the local ethical guidelines (as stipulated by the Declaration of Helsinki). Written informed consent was obtained from the participants. The protocol was approved by the review board of Sun Yat-Sen University.

## Results

### Icaritin Suppresses Tumor Growth and Prolongs Survival of Mice Bearing HCC Tumors

**Figure 1:**
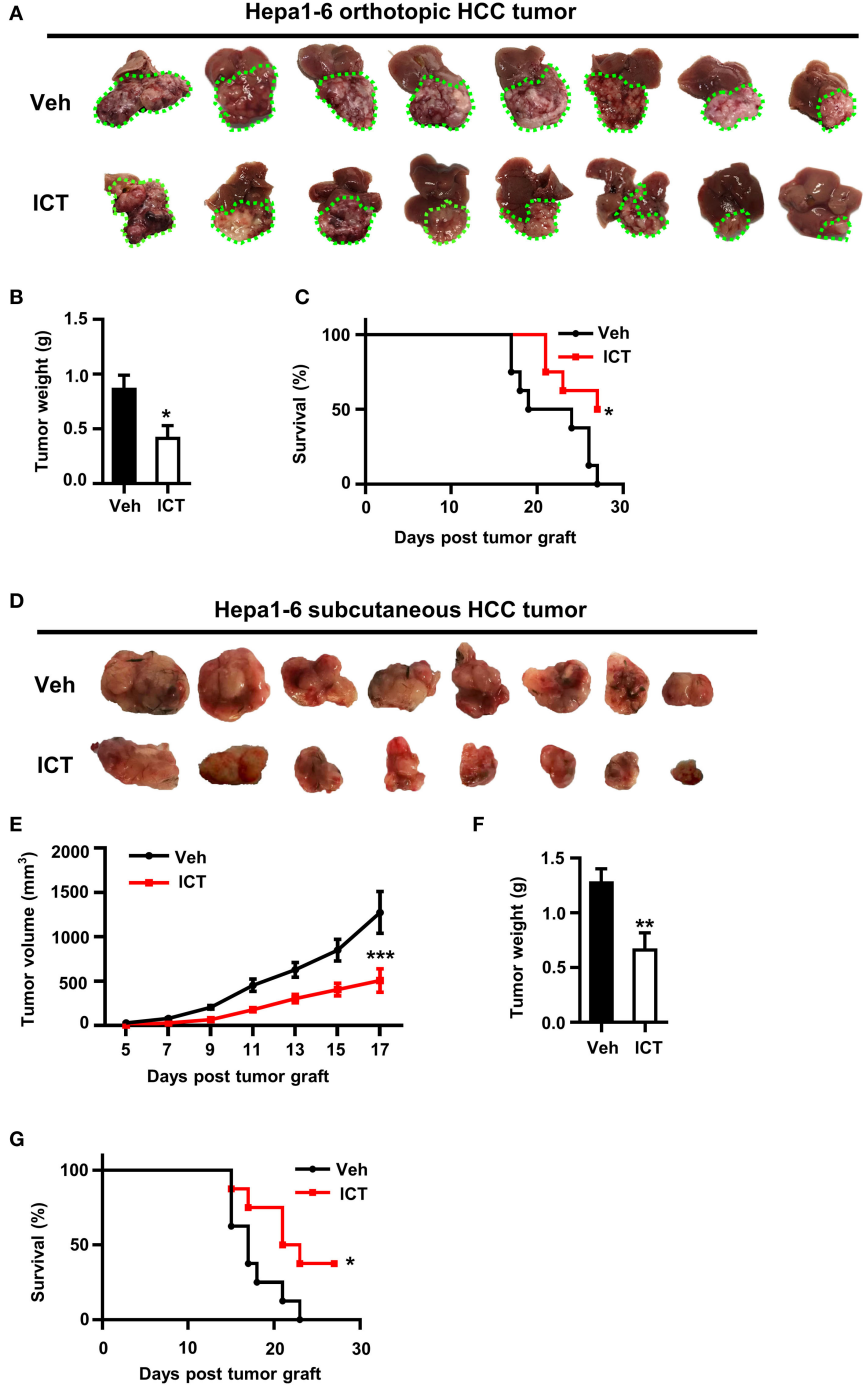
Icaritin inhibits the growth of orthotopic and subcutaneous Hepa tumors. **(A)** Images of orthotopic tumors 26–28 days after inoculation (green dotted line indicates tumor margins). **(B)** Orthotopic tumor weights compared by Student's *t*-test. **(C)** Kaplan-Meier curves indicating the survival of mice with orthotopic tumors treated with and without icaritin. **(D)** Images of subcutaneous tumors 26–28 days after inoculation. **(E)** Mean subcutaneous tumor volume was recorded every other day and analyzed by two-way ANOVA corrected by Bonferroni's test. **(F)** Subcutaneous tumor weights compared by Student's *t*-test. **(G)** Kaplan-Meier curves indicating the survival of mice with subcutaneous tumors treated with and without icaritin. Data were pooled from two experiments and *n* = 8 mice per group. *P < 0.05, **P < 0.01, ***P < 0.001. Hepa, Hepa1-6 cells; ICT, icaritin; Veh, Vehicle; HCC, hepatocellular carcinoma.

To investigate the anti-tumor potential of icaritin *in vivo*, a murine Hepa1-6 (Hepa) cell-based orthotopic HCC tumor model was adopted, which reproduces many key pathological features of human HCC (21, 33). After 3 weeks of daily icaritin treatment at 70 mg/kg icaritin, all mice were sacrificed and tumors were collected. Tumor size and weight were significantly reduced following icaritin treatment (Figures 1A,B). Furthermore, for all mice in the control group, mortality occurred within 27 days, whereas 50% of mice treated with icaritin survived for more than 27 days (Figure 1C).

The anti-tumor activity of icaritin was also examined using a subcutaneous Hepa cell implant model, commonly used for drug screening (33). Icaritin treatment significantly suppressed tumor growth, reduced tumor weight, and improved survival (Figures 1D,F,G). Icaritin markedly suppressed tumor growth at various time points (Figure 1E). It should be noted that the mice treated with icaritin and the control group did not show any significant difference in the body weight (Supplementary Figure 1). Furthermore, icaritin treatment did not affect the apoptotic rate, proliferation, or the protein level of STAT3 in Hepa cells cultured *in vitro* (Supplementary Figure 2).

### Icaritin Increases Both the Total Number and Active Tumor-Infiltrating Cytotoxic T Lymphocytes (CTLs) in Mice Challenged With Hepa Cells

**Figure 2:**
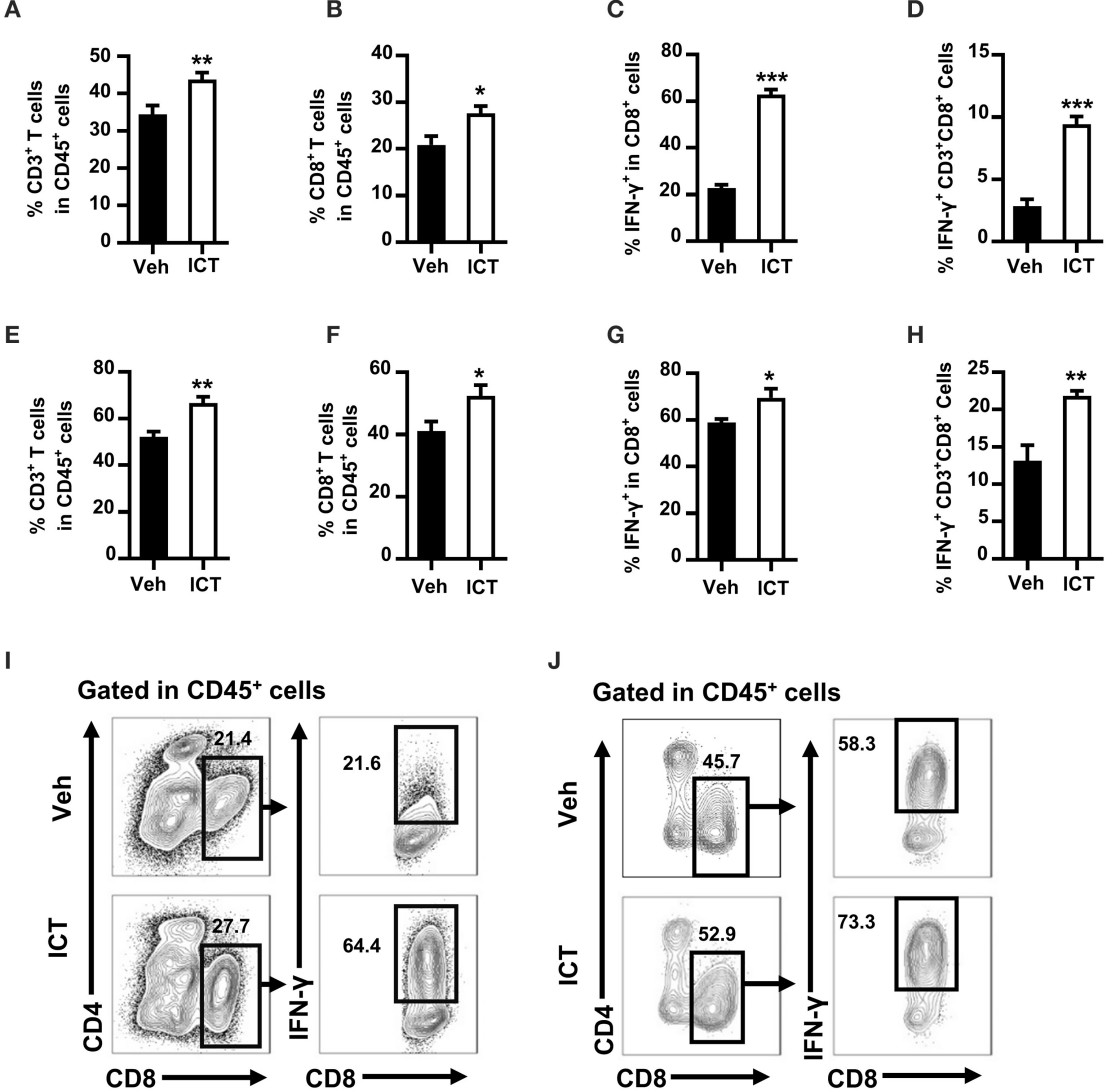
Icaritin increases both the number and activation status of tumor-infiltrating CTLs in Hepa mice. The frequencies of tumor-infiltrating CD3^+^ T cells **(A)** CD3^+^CD8^+^ T cells **(B)** IFN-γ^+^CD3^+^CD8^+^ cells in CD3^+^CD8^+^ T cells **(C)** and IFN-γ^+^CD3^+^CD8^+^ cells in CD45^+^ cells **(D)** from orthotopic Hepa tumors. The frequencies of tumor-infiltrating CD3^+^ T cells **(E)** CD3^+^CD8^+^ T cells **(F)** IFN-γ^+^CD3^+^CD8^+^ cells in CD3^+^CD8^+^ T cells **(G)** and IFN-γ^+^CD3^+^CD8^+^ cells in CD45^+^ cells **(H)** from subcutaneous Hepa tumors. Representative cytometric plots of tumor-infiltrating IFN-γ^+^CD3^+^CD8^+^ cells from orthotopic **(I)** and subcutaneous **(J)** Hepa tumors. Numbers in the flow cytometric plots indicate the proportions of the gated cell populations. Data were pooled from two experiments and *n* = 8 mice per group. *P < 0.05, **P < 0.01, ***P < 0.001. Hepa, Hepa1-6 cells; ICT, icaritin; Veh, Vehicle.

Cumulative studies have demonstrated that an efficient induction of CTLs is essential for anti-tumor therapy (7). To determine whether icaritin could induce CTLs production in the tumor microenvironment, flow cytometry was used to analyze the composition of tumor-infiltrating immune cell populations in the orthotopic Hepa mice. Icaritin treatment influenced the percentages of T cells among the leukocyte subsets (CD45^+^ cells). Icaritin enriched the proportion of tumor-infiltrating T cells in the orthotopic Hepa mice compared to that in the control (43.8 ± 0.7% vs. 34.5 ± 1.1%; Figure 2A). In particular, icaritin increased CD3^+^CD8^+^ T cell frequency in the tumor tissue compared to that in the control (27.6 ± 0.8% vs. 20.8 ± 1.1%; Figures 2B,I).

To examine the activation status of tumor-infiltrating CD8^+^ T cells, the expression of IFN-γ by CD3^+^CD8^+^ T cells in the tumor tissue was analyzed. Icaritin treatment increased IFN-γ^+^ CD3^+^CD8^+^ CTLs by almost 3-fold in CD3^+^CD8^+^ T cells (62.8 ± 2.3% vs. 22.7 ± 1.6%; Figures 2C,I) and in CD45^+^ cells (9.4 ± 0.7% vs. 2.8 ± 0.6%; Figure 2D). A similar effect was observed in subcutaneous tumor tissue of Hepa mice, albeit less pronounced (Figures 2E–H,J). It should be noted that icaritin treatment did not alter the frequencies of tumor-infiltrating B cells or NK cells (Supplementary Figures 3C,D). In addition, there was no significant effect of icaritin treatment on the percentage of regulatory T cells in the tumor tissue in either model when compared with the control (Supplementary Figures 3C,D). These data indicate that icaritin treatment may stimulate an anti-tumor response by selectively increasing the number and activation status of CTLs in the tumor tissue.

### Icaritin Reduces Both the Number and Activity of Tumor-Infiltrating PMN-MDSCs

**Figure 3:**
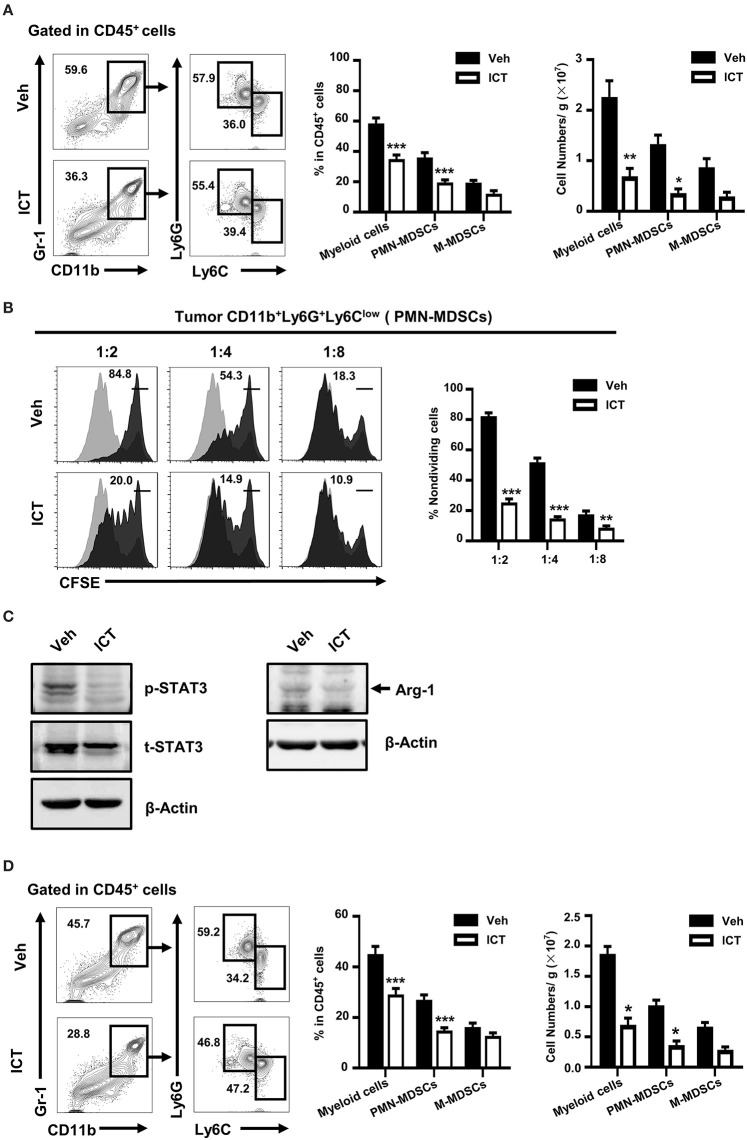
Icaritin reduces both the number and activation status of tumor-infiltrating PMN-MDSCs. **(A)** Frequency and number of tumor-infiltrating myeloid cells (CD11b^+^Gr-1^+^), PMN-MDSCs (CD11b^+^Gr-1^+^Ly6G^+^Ly6C^low^), and M-MDSCs (CD11b^+^Gr-1^+^Ly6G^−^Ly6C^high^) orthotopic Hepa mice. **(B)** Immunosuppressive activity of orthotopic tumor-infiltrating PMN-MDSCs on *in vitro* T cell proliferation (stimulated with anti-CD3- and anti-CD28) at the ratios of 1:2, 1:4, and 1:8. **(C)** Western blots indicating Arg-1 protein expression and STAT3 activation in PMN-MDSCs isolated from tumors of orthotopic Hepa mice. **(D)** Frequency and number of tumor-infiltrating myeloid cells (CD11b^+^Gr-1^+^), PMN-MDSCs (CD11b^+^Gr-1^+^Ly6G^+^Ly6C^low^), and M-MDSCs (CD11b^+^Gr-1^+^Ly6G^−^Ly6C^high^) in subcutaneous Hepa mice. Numbers in the flow cytometric plots indicate the proportions of the gated cell populations. Differences between groups were analyzed using two-way ANOVA corrected by Bonferroni's test. Data were pooled from two experiments and *n* = 8 mice per group. *P < 0.05, **P < 0.01, ***P < 0.001. Hepa, Hepa1-6 cells; ICT, icaritin; Veh, Vehicle; Arg-1, Arginase-1; M-MDSCs, mononuclear myeloid-derived suppressor cells; PMN-MDSCs, polymorphonuclear myeloid-derived suppressor cells; p, phosphorylated; t, total.

Tumor-associated myeloid cells, including MDSCs, regulate the immunosuppressive activity of the tumor microenvironment, which is an obstacle in the success of cancer therapy (15, 16). MDSCs can be classified into two subpopulations: PMN-MDSCs and monocytic MDSCs (M-MDSCs). In murine systems, PMN-MDSCs are defined as CD11b^+^Ly6G^+^Ly6C^low^, and M-MDSCs are defined as CD11b^+^Ly6G^−^Ly6C^high^. Depletion of MDSC frequency or abrogation of the differentiation or immunosuppressive function of these cells, may potentially enhance various therapeutic strategies by supporting the anti-tumor immune response (21, 31, 34, 35). To investigate whether this mechanism contributes to the observed anti-tumor activity of icaritin, we used flow cytometry to evaluate the composition of tumor-infiltrating myeloid cells derived from orthotopic Hepa mice. As shown in Figure 3A, the frequency of tumor-infiltrating CD11b^+^Gr-1^+^ myeloid cells in the orthotopic Hepa mice was significantly lower in icaritin-treated mice compared to that in the control (35.5 ± 3.2% vs. 58.8 ± 7.5%). Icaritin also caused a marked reduction in the number of tumor myeloid cells (2.3 ± 0.3 × 10^7^ vs. 0.7 ± 0.2 × 10^7^). Icaritin did not significantly affect M-MDSC frequency, but significantly decreased the proportion (36.0 ± 3.2% vs. 19.5 ± 1.7%; Figure 3A) and number (1.3 ± 0.2 × 10^7^ vs. 0.3 ± 0.1 × 10^7^; Figure 3A) of PMN-MDSCs in the tumor tissues.

We next investigated whether icaritin affects the immunosuppressive function of tumor-infiltrating MDSCs. Icaritin impaired the suppressive effect of tumor-infiltrating PMN-MDSCs on the proliferation of T cells (Figure 3B). It has been suggested that the transcription factor, STAT3, regulates the immunosuppressive activity and accumulation of MDSCs in the tumor microenvironment (36, 37). Arginase-1 (Arg-1) is an immunosuppressive factor expressed by MDSCs that suppresses CD8^+^ T cell function and anti-tumor immune responses (38). Icaritin reduced the expression of Arg-1 in tumor-infiltrating PMN-MDSCs from the tumors of orthotopic Hepa mice, and inhibited STAT3 activation in PMN-MDSCs (Figure 3C). These results demonstrate that icaritin not only repressed the frequency and number of PMN-MDSCs *in vivo* but also attenuated their T cell suppressive activity. Similar to its effects in the orthotopic HCC tumor model, icaritin downregulated the frequency of myeloid cells and PMN-MDSCs in subcutaneous tumors of Hepa mice (Figure 3D). However, there was no significant difference in the macrophage number in either model (Supplementary Figures 3C,D). These results suggest that icaritin decreases tumor infiltration of MDSCs as well as their immunosuppressive activity.

### Icaritin Reduces PMN-MDSC Frequency and Increases CTL Frequency in the Spleen

**Figure 4:**
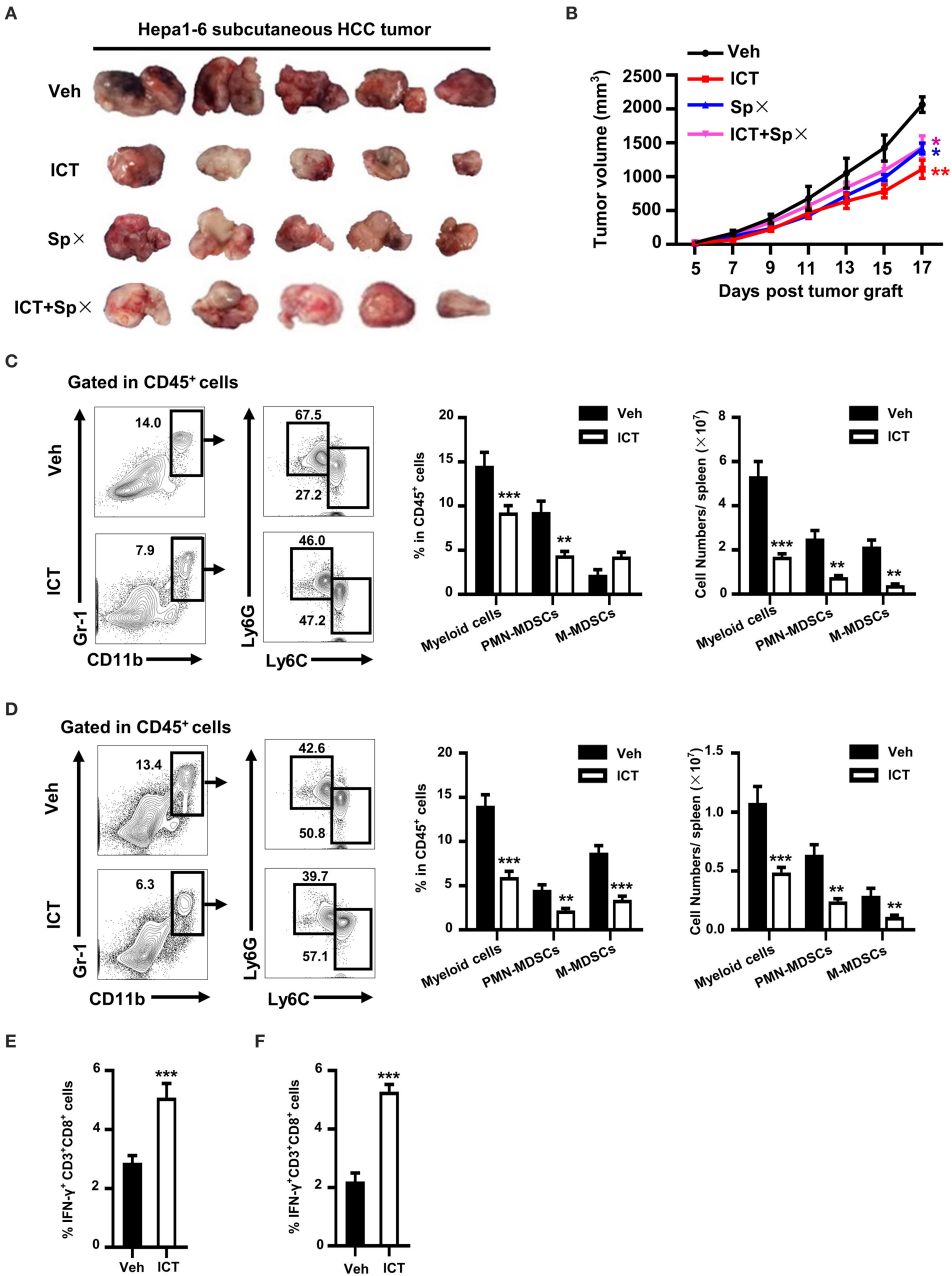
Effects of icaritin on splenic PMN-MDSCs and CTLs. Images of subcutaneous tumors 26 days after inoculation **(A)**. Mean tumor volume of subcutaneous Hepa tumor-bearing mice subjected to splenectomy with or without icaritin treatment **(B)**. Differences between groups were examined for statistical significance by two-way ANOVA, and corrected by Bonferroni's test. *P < 0.05 and **P < 0.01 compared with “Veh” group. Frequencies and total numbers of splenic myeloid cells, PMN-MDSCs and M-MDSCs in orthotopic **(C)** and subcutaneous **(D)** Hepa mice. Frequency of splenic IFN-γ^+^CD3^+^CD8^+^ cells in CD45^+^ cells from orthotopic **(E)** and subcutaneous **(F)** Hepa mice. Numbers in the flow cytometric plots indicate the proportions of the gated cell populations. Differences between groups were analyzed using two-way ANOVA corrected by Bonferroni's test **(C**, **D)**, or examined for statistical significance by Student's *t*-test **(E**, **F)**. Data were pooled from two experiments and *n* = 8 mice per group. **P < 0.01, ***P < 0.001. SPx, splenectomy; Hepa, Hepa1-6 cells; ICT, icaritin; Veh, Vehicle; M-MDSCs, mononuclear myeloid-derived suppressor cells; PMN-MDSCs, polymorphonuclear myeloid-derived suppressor cells; CTLs, cytotoxic T lymphocytes.

Since icaritin is known to have anti-tumor function and reduce MDSC (28), we utilized the anti Gr-1 antibody to verify whether the anti-HCC function of icaritin is through targeting MDSC. Depletion of MDSCs abrogated the inhibitory effect on tumor growth of icaritin (Supplementary Figures 4A,B). It has been shown that the spleen is a major source of MDSCs, as it accommodates myeloid-biased hematopoiesis to facilitate the generation of functional MDSCs in a tumor-bearing host (21, 35). We noticed that splenectomy abrogate the anti-tumor effect of icaritin (Figures 4A,B), which suggested that the anti-HCC function of icaritin is through targeting MDSC generation in spleen. Given the aforementioned findings that icaritin downregulates the accumulation of MDSCs in the tumor tissue, we next assessed whether icaritin affects the accumulation of MDSCs in the spleen. Icaritin significantly decreased the number of splenic myeloid cells and PMN-MDSCs in orthotopic Hepa mice (Figure 4C). In CD45^+^ cells, icaritin treatment resulted in decreased frequency of myeloid cells and PMN-MDSCs (14.6 ± 1.5% and 9.3 ± 1.2%, vehicle, vs. 9.3 ± 0.8% and 4.4 ± 0.4%, icaritin). Icaritin reduced myeloid cell and PMN-MDSC number from 5.3 ± 1.9 × 10^7^ and 2.5 ± 1.0 × 10^7^ to 1.7 ± 0.4 × 10^7^ and 0.8 ± 0.2 × 10^7^ per spleen, respectively (Figure 4C). Similarly, icaritin reduced the proportion and number of PMN-MDSCs and myeloid cells in the spleens of Hepa mice with subcutaneous tumor (Figure 4D). Similar to the results found in the tumor tissue, icaritin had a negligible effect on splenic M-MDSCs.

As MDSCs inhibit antigen-specific CD8^+^ T cell function and icaritin reduces MDSC number, we hypothesized that T cell function would correspondingly recover in icaritin-treated mice with HCC. To test this hypothesis, IFN-γ production was analyzed as a readout of T cell anti-tumor activity. A significantly higher proportion of splenocytes isolated from mice treated with icaritin were IFN-γ-producing CD8^+^ T cells compared to that in the control mice in both models (Figures 4E,F). These results suggest that icaritin treatment can promote CTLs function by inhibiting MDSC production in the spleen of mice with HCC.

### Icaritin Decreases Accumulation of Myeloid-Biased Hematopoietic Stem and Progenitor Cells (HSPCs) in the Spleen

**Figure 5:**
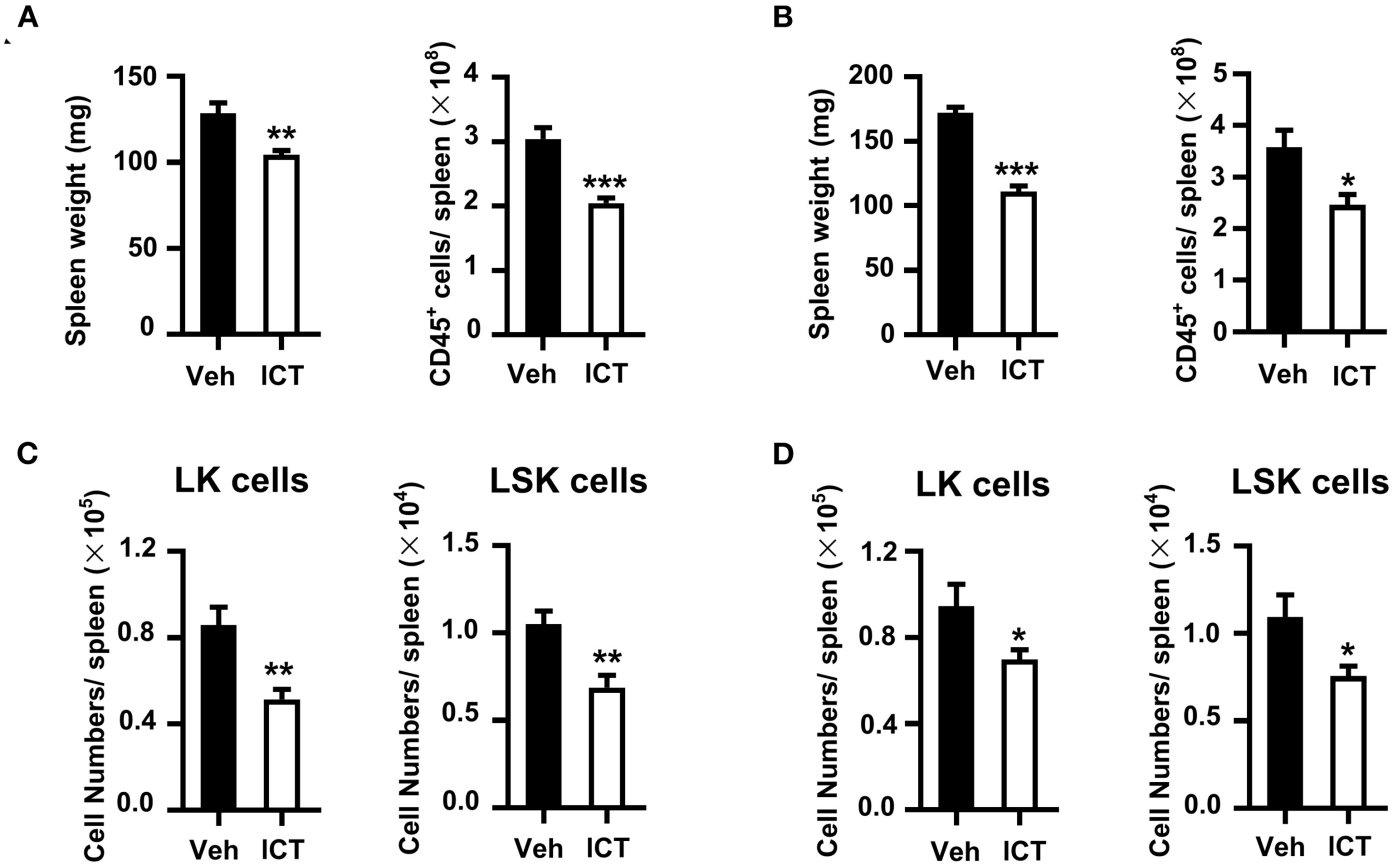
Icaritin reduces the accumulation of HSPCs in the spleen of Hepa mice. Spleen weight and total cell number from orthotopic **(A)** and subcutaneous **(B)** Hepa mice. Numbers of splenic LSK and LK cells from orthotopic **(C)** and subcutaneous **(D)** Hepa mice. Differences between groups were examined for statistical significance by Student's *t*-test. Data were pooled from two experiments and *n* = 8 mice per group. *P < 0.05, **P < 0.01, ***P < 0.001. HSPCs, hematopoietic stem and progenitor cells; Hepa, Hepa1-6 cells; ICT, icaritin; Veh, Vehicle; LSK, Lin^lo/−^Sca-1^+^c-Kit^hi^; LK, Lin^lo/−^Sca-1^−^c-Kit^hi^.

Our previous study showed that cancer facilitates the accumulation of myeloid biased HSPCs in the spleen to generate functional MDSCs by EMH (21). In this study, we observed that icaritin treatment resulted in reduced spleen weight and a significant decrease in total spleen cell number in the Hepa mice with orthotopic tumor compared to that in the control (Figure 5A).

This may indicate that icaritin suppressed splenic EMH in this model. To investigate this further, the influence of icaritin on the composition of splenic HSPCs was assessed. LSK (Lin^lo/−^Sca-1^+^c-Kit^hi^) and LK (Lin^lo/−^Sca-1^−^c-Kit^hi^) cells are two subpopulations of HSPCs, which could develop into myeloid cells in the spleen of tumor-bearing mice (21). The spleens of orthotopic Hepa mice treated with icaritin exhibited a significant reduction of 45% in the number of splenic LK cells per spleen compared to the control (0.9 ± 0.1 × 10^5^ vs. 0.5 ± 0.1 × 10^5^; Figure 5C). Icaritin also caused a marked decrease in splenic LSK cell number per spleen (1.1 ± 0.1 × 10^4^ vs. 0.7 ± 0.1 × 10^4^; Figure 5C). In contrast, icaritin did not affect the accumulation of LSK or LK cells in the bone marrow (Supplementary Figure 5A). Similar results were obtained in Hepa mice with subcutaneous tumor (Figures 5B,D; Supplementary Figure 5B). Thus, these results support the hypothesis that icaritin decreases tumor-induced splenic myeloid biased-hematopoiesis to inhibit MDSC generation.

### Icaritin Impairs the Generation of Human PMN-MDSCs

**Figure 6:**
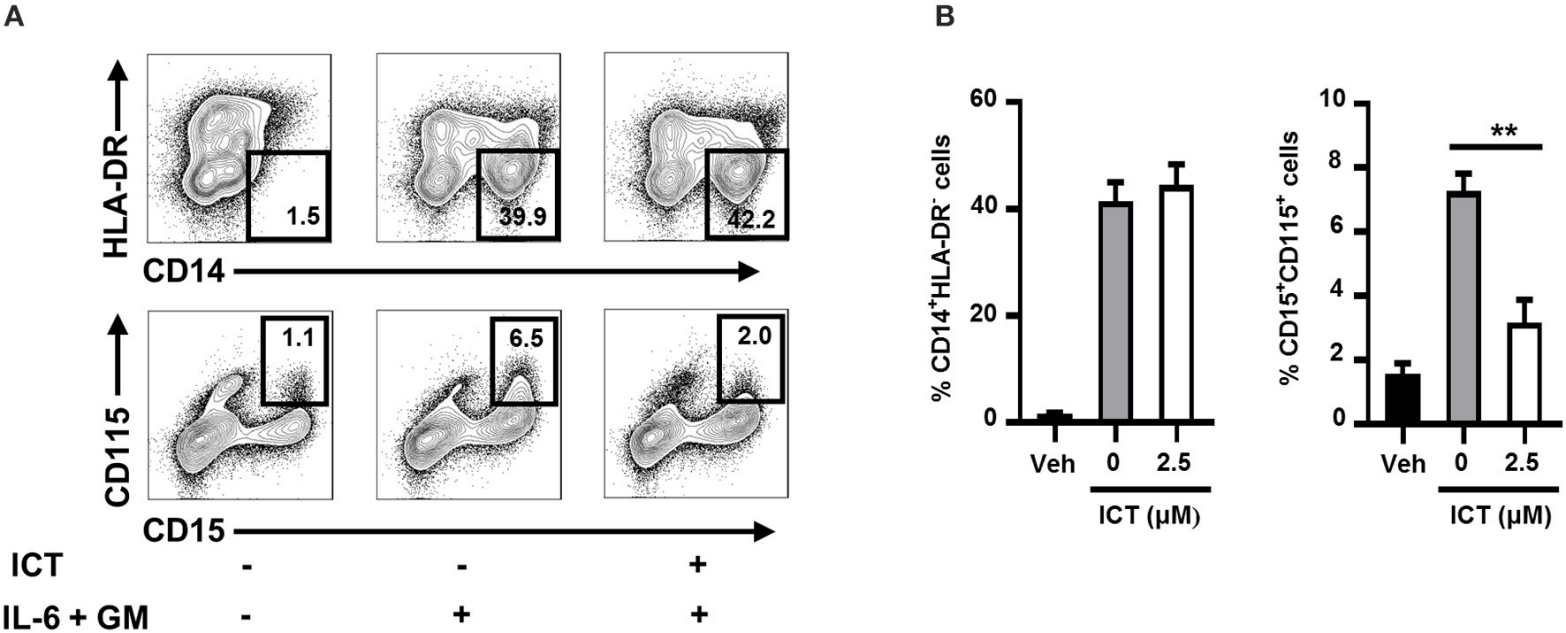
Icaritin inhibits the generation of human PMN-MDSCs *in vitro*. Freshly isolated human CD34^+^ cells from cord blood mononuclear cells were cultured in hematopoietic stem cell expansion media for 8–10 days. The expanded cells were cultured with combined IL-6 and G-CSF in complete medium with 0.1% DMSO (vehicle) or icaritin (2.5 μM) for 3 days. **(A)** Flow cytometric analysis of CD115 and HLA-DR expression on CD14^+^ or CD15^+^ cells. **(B)** Frequencies of PMN-MDSCs (CD15^+^CD115^+^) and M-MDSCs (CD14^+^HLA-DR^−^). Differences between groups were analyzed using one-way ANOVA, and corrected by Dunnett's test. Data are representative of three experiments. **P < 0.01. Veh, Vehicle; ICT, Icaritin.

The results described collectively indicate that icaritin inhibits MDSC generation in mice. We next sought to determine whether icaritin could affect the formation of human MDSCs. Human M-MDSCs are typically phenotyped as CD14^+^HLA-DR^low/−^ (39), with CD115 as a distinct surface marker for the activation of the MDSC suppressive program (19). A short-term culture model using cord blood-derived CD34^+^ cells was utilized to rapidly generate MDSCs *in vitro*. We confirmed that icaritin markedly decreased the proportion of activated human PMN-MDSCs (CD115^+^CD15^+^), but did not affect the generation of M-MDSCs (CD14^+^HLA-DR^low/−^; Figures 6A,B). The findings in mice and human suggest that icaritin may attenuate the generation of PMN-MDSCs by regulating the differentiation of HSPCs.

### Icaritin Synergistically Enhances the Anti-PD-1 Efficacy

**Figure 7:**
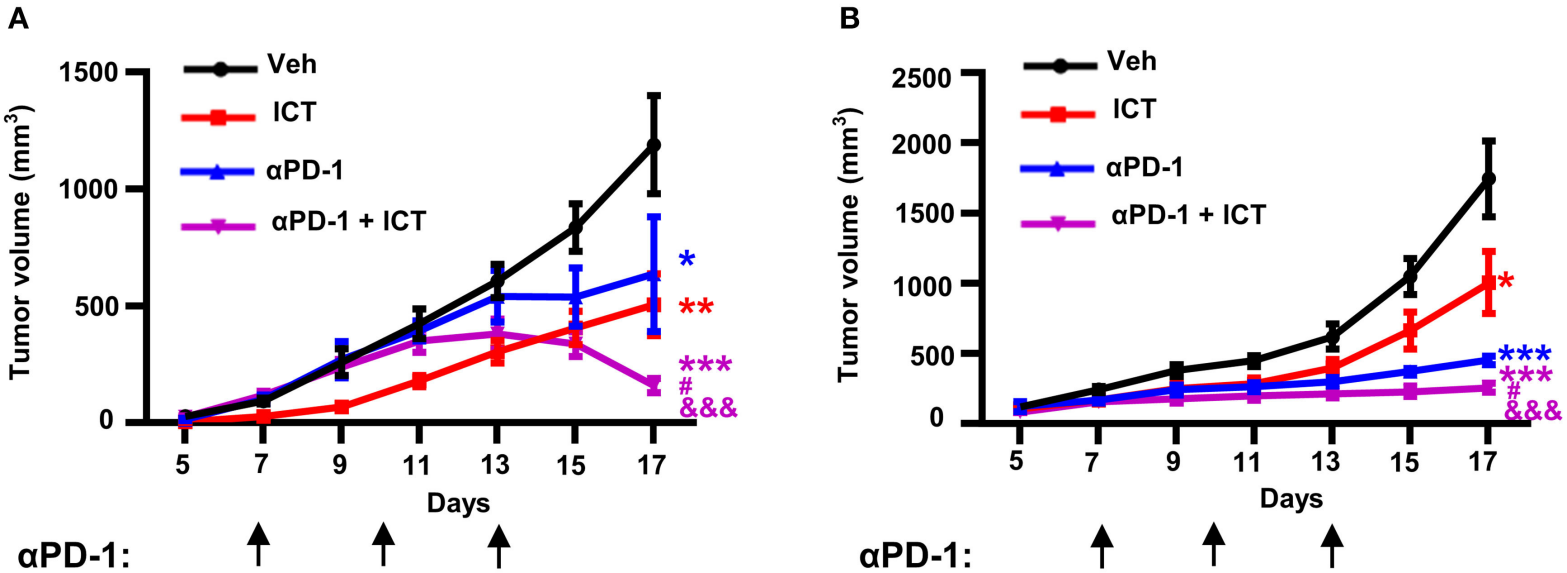
Icaritin synergistically enhances anti-PD-1 efficacy in HCC mice. Mean tumor volume of subcutaneous Hepa **(A)** and subcutaneous H22 **(B)** tumor-bearing mice with and without anti-PD-1 and icaritin treatment. Differences between groups were examined for statistical significance by two-way ANOVA, and corrected by Bonferroni's test. *P < 0.05, **P < 0.01, and ***P < 0.001 compared with “Veh” group; ^#^*P* < 0.05 compared with “αPD-1” group; ^&&&^*P* < 0.001 compared with “ICT” group. Hepa, Hepa1-6 cells; ICT, icaritin; Veh, Vehicle; αPD-1, anti-PD-1.

Immunosuppressive myeloid cells contribute toward the resistance to immune checkpoint blockade therapy by suppressing T cell function (16). It has been previously described that the abrogation of splenic EMH (21) or selective targeting of MDSCs (16) are sufficient to synergistically enhance the therapeutic efficacy of immune checkpoint blockade. Given that icaritin attenuated splenic EMH and MDSC generation in tumor-bearing mice, we hypothesized that icaritin may promote the efficacy of immunotherapy. Furthermore, icaritin upregulated the expression of PD-L1 on tumor-infiltrating and splenic PMN-MDSCs (Supplementary Figure 6), which may indicate an opportunity for combined therapy using immune checkpoint blockade. As shown in Figure 7A, anti-PD-1 monotherapy had a modest effect on tumor growth in orthotopic Hepa mice. However, icaritin synergistically enhanced the therapeutic efficacy of anti-PD-1. In addition, the synergistic effect of icaritin and anti-PD-1 combinational treatment was verified in subcutaneous H22 tumor-bearing BALB/c mice (Figure 7B). Together, these findings demonstrate that icaritin may be applied together with immune checkpoint blockade therapy for HCC treatment.

## Discussion

Recent studies have suggested that splenic EMH is involved in the course of tumor development as well as cancer therapeutic responses (21, 40–42). Therapeutic interventions targeting tumor-associated EMH have become attractive strategies for cancer treatment. In this study, icaritin induced tumor regression and significantly prolonged the survival of mice bearing orthotopic or subcutaneous HCC tumors. Icaritin significantly reduced the accumulation of tumoral and splenic MDSCs, and increased the number and activity of CTLs, possibly delaying disease progression. This may be due to the downregulation of tumor-associated splenic EMH induced by icaritin, and the subsequent generation of functional MDSCs. In addition, icaritin enhanced the therapeutic effect of anti-PD-1 immune checkpoint blockade therapy in mice with HCC. These findings indicate that icaritin may serve as a novel immunomodulatory drug to attenuate immunosuppression in the tumor microenvironment by controlling the generation of functional MDSCs in HCC tumors.

Accumulating evidence indicates that icaritin is a promising anti-tumor agent in various types of cancers. Icaritin exerts its anti-tumor activity via a multitude of cellular targets and through a variety of pathways, including the inhibition of cell growth, induction of tumor cell apoptosis, and immunomodulation (23, 29). The present study provides evidence that the anti-tumor activity of icaritin observed in HCC tumors may be affected via its modulatory effects on the immune-microenvironment of tumor tissues rather than its direct cytotoxic effects on tumor cells. In the present study, icaritin treatment did not induce apoptosis or inhibit the growth of Hepa cells *in vitro*. Furthermore, in HCC tumor cells, icaritin did not affect the expression of STAT3, known to be required for cytotoxicity induced by icaritin (43, 44). These results indicate that icaritin had no direct cytotoxic effect on tumor cells, suggesting that icaritin treatment likely delays tumor progression via an indirect immunomodulatory mechanism. This is supported by our findings which showed that icaritin increased the frequency of tumor-infiltrating and splenic IFN-γ-producing CD8^+^ T cells *in vivo*, thereby indicating that the anti-tumor activity of icaritin is effected by immunomodulation in mice with HCC. This is in accordance with a previous report that advanced HCC patients with increased circulating plasma levels of IFN-γ benefit from icaritin treatment (30).

Given that MDSCs suppress the anti-tumor response, they act as a hindrance to the success of cancer therapy. Current therapeutic strategies targeting MDSCs include elimination, functional inhibition of the MDSC suppressive activity and skewing of myelopoiesis away from the generation of MDSCs (33, 45). Recent studies have suggested that abnormal splenic EMH is responsible for generating immunosuppressive MDSCs in tumors (21, 46). Therefore, targeted abrogation of splenic EMH may potentiate anti-cancer therapy (20, 21). The decrease in the number of splenic LSK and LK HSPCs with icaritin was associated with a corresponding decrease in MDSC frequency in the present study. This was supported by the inhibition of PMN-MDSC generation from HSPCs with icaritin treatment *in vitro*. A possible conclusion is that icaritin inhibited the generation of MDSCs by blocking splenic EMH in tumor-bearing mice. Reduced Arg-1 expression in PMN-MDSCs from tumor tissue *in vivo*, and reduced M-CSFR expression on human cord blood-derived MDSCs *in vitro* (30) suggest that icaritin may exert its anti-tumor activity via impairing the MDSCs through multiple mechanisms of action.

Immune checkpoint therapies targeting PD-1 and PD-L1 have achieved remarkable clinical responses in various types of cancer, including HCC (6). However, the objective response rates are still limited. Based on our findings that icaritin possesses immune-regulatory properties, we speculated that icaritin in combination with immunotherapy strategies could enhance the therapeutic efficacy of immune checkpoint blockade. Anti-PD-1 and anti-PD-L1 monoclonal antibodies can relieve immunosuppression and restore anti-tumor immune responses (47). We demonstrated that the anti-tumor immune response induced by anti-PD-1 monoclonal antibody is indeed enhanced by icaritin. Icaritin also upregulated PD-L1 expression on tumoral and splenic PMN-MDSCs. It has been previously demonstrated that PD-L1 expression on myeloid cells is associated with an immune-activated microenvironment (48, 49). However, the underlying mechanism of PD-L1 regulation by icaritin remains unclear. Whether icaritin regulates PD-L1 expression directly or through the attenuation MDSC activity requires further investigation.

In summary, this work describes a novel anti-tumor mechanism of icaritin that involves the targeting of EMH to inhibit the generation and activation of MDSCs, thus reshaping the tumor immune microenvironment and promoting cytotoxic T cell-mediated tumor regression. We demonstrated that icaritin exerts potent immunomodulatory and anti-tumor effects, suggesting that it may be developed into a novel adjuvant or even stand-alone therapeutic agent for the effective treatment of HCC.

## Data Availability Statement

The raw data supporting the conclusions of this article will be made available by the authors, without undue reservation.

## Ethics Statement

The studies involving human participants were reviewed and approved by local ethical guidelines (as stipulated by the Declaration of Helsinki). The patients/participants provided their written informed consent to participate in this study. The animal study was reviewed and approved by IACUC of the Sun Yat-sen University Cancer Center.

## Author Contributions

LL, LZ, and KM was the principal investigator and designed the research. HT, ML, YW, SL, YX, and BY performed the experiments. HT, ML, YW, LL, and LZ analyzed the results and wrote the manuscript. All authors read and commented on the manuscript.

## Conflict of Interest

BY and KM are employees of Beijing Shenogen Biomedical Ltd. The remaining authors declare that the research was conducted in the absence of any commercial or financial relationships that could be construed as a potential conflict of interest.

## Abbreviations

HCC: hepatocellular carcinoma
EMH: extramedullary hematopoiesis
MDSCs: myeloid-derived suppressor cells
CTLs: cytotoxic T cells
PMN-MDSCs: polymorphonuclear MDSCs
M-MDSCs: monocytic MDSCs
Arg-1: arginase-1
Veh: vehicle
ICT: icaritin
HSPCs: hematopoietic stem and progenitor cells.
