# Supplementary figures and images for: Icaritin Induces Anti-tumor Immune Responses in Hepatocellular Carcinoma by Inhibiting Splenic Myeloid-Derived Suppressor Cell Generation
Source: Front Immunol. 2021 Feb 26;12:609295. doi: 10.3389/fimmu.2021.609295 (PMC7952329; doi:10.3389/fimmu.2021.609295)

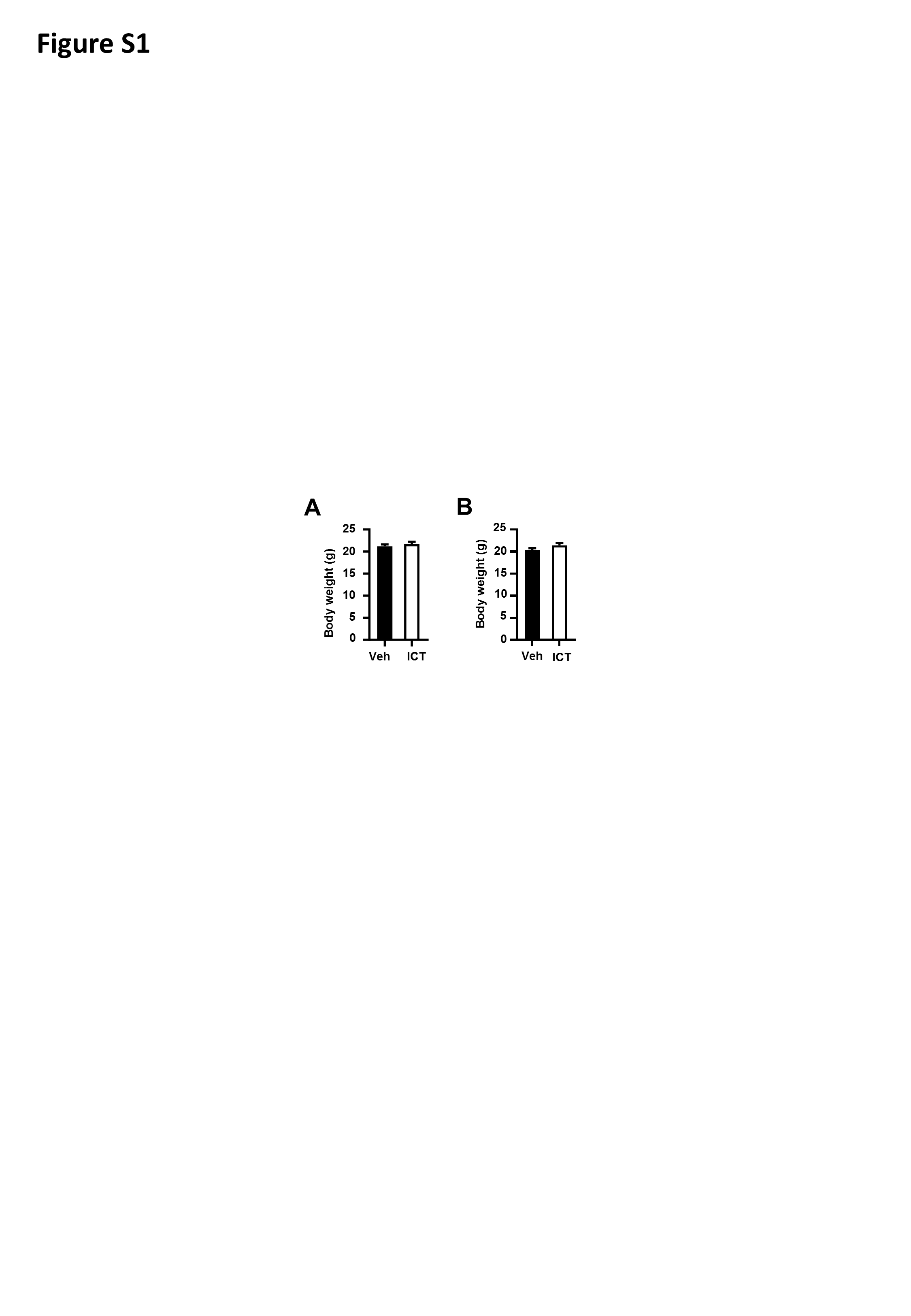

Supplement: Supplementary file 1 [file Image_1.tif]

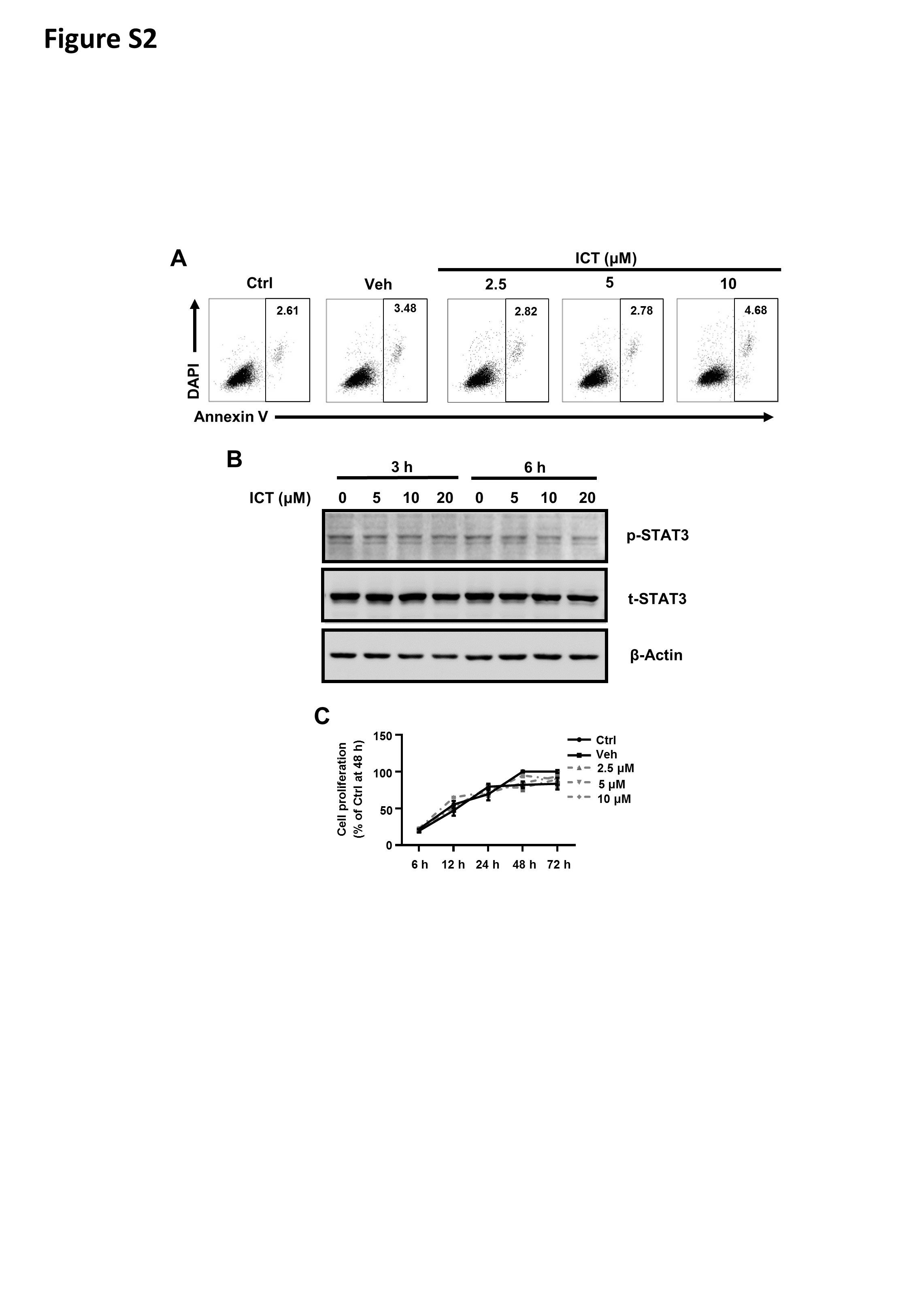

Supplement: Supplementary file 2 [file Image_2.tif]

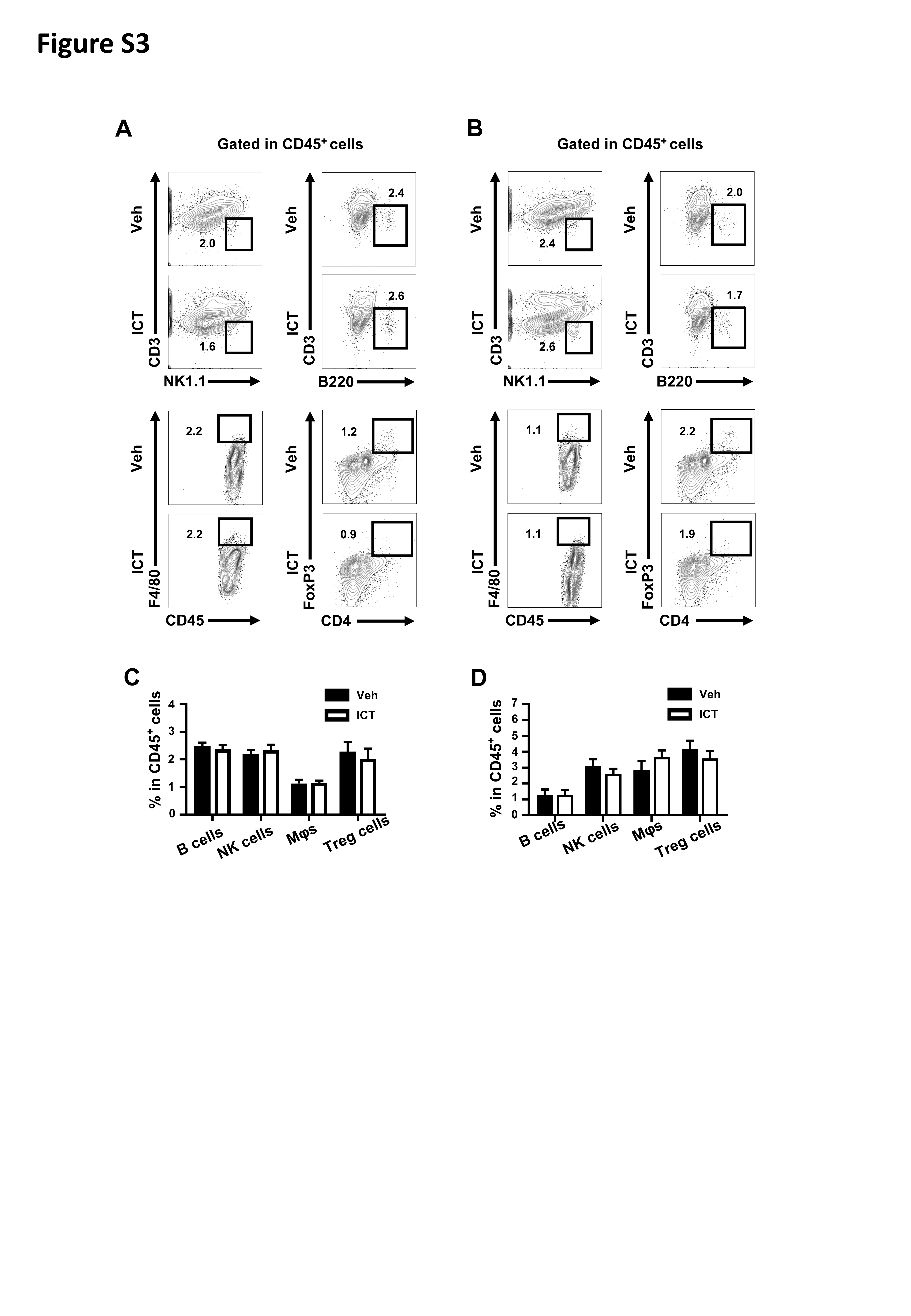

Supplement: Supplementary file 3 [file Image_3.tif]

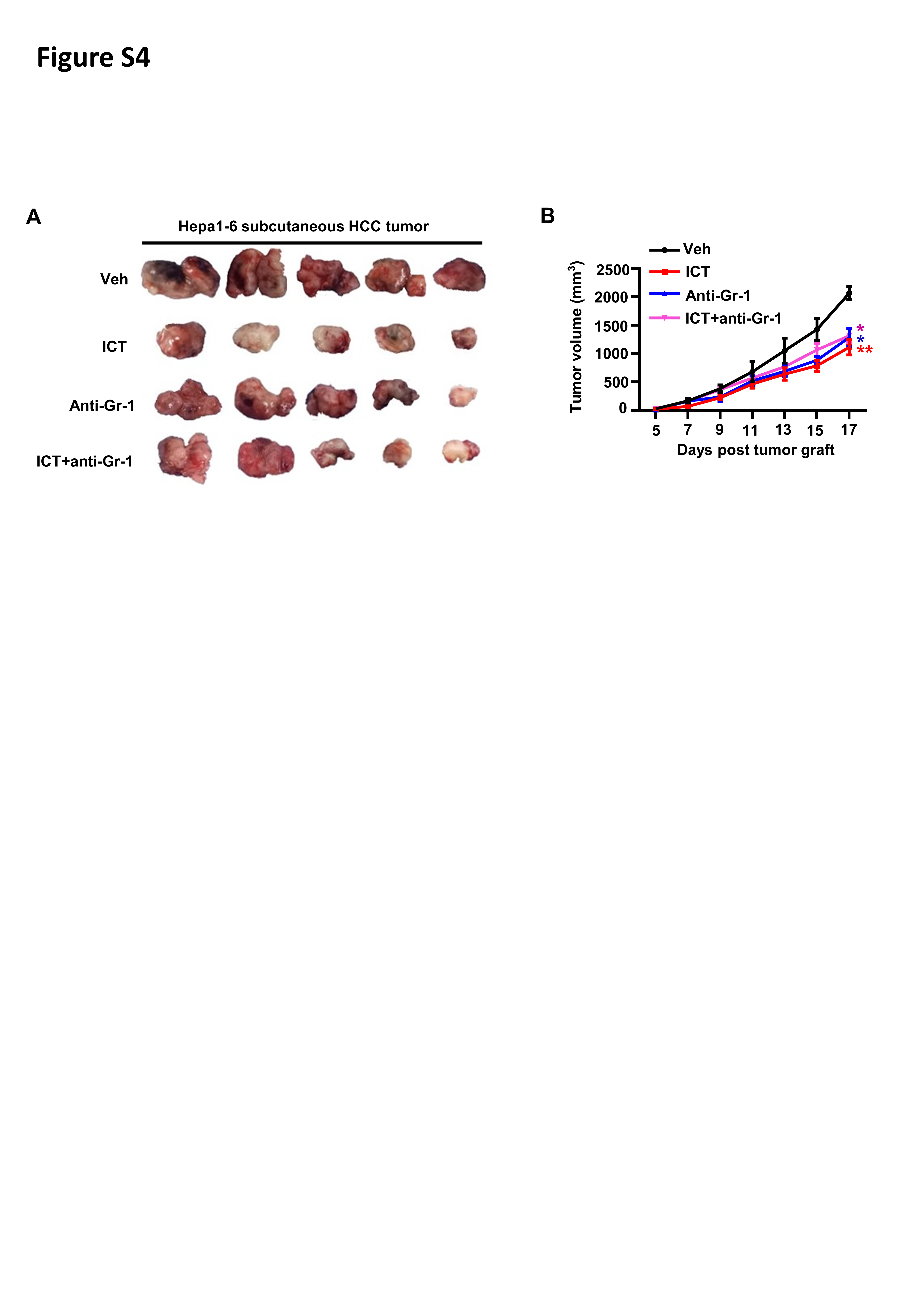

Supplement: Supplementary file 4 [file Image_4.tif]

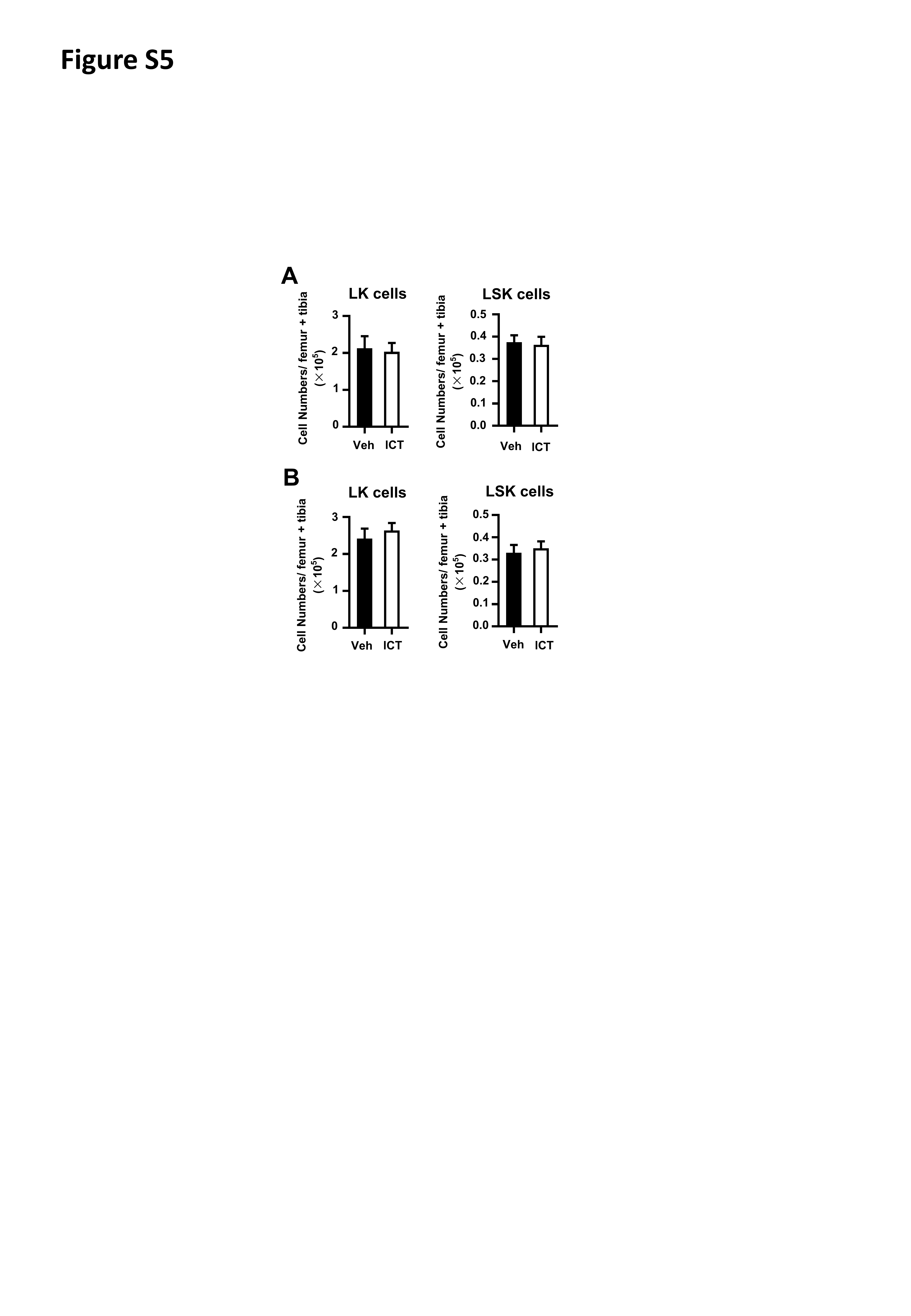

Supplement: Supplementary file 5 [file Image_5.tif]

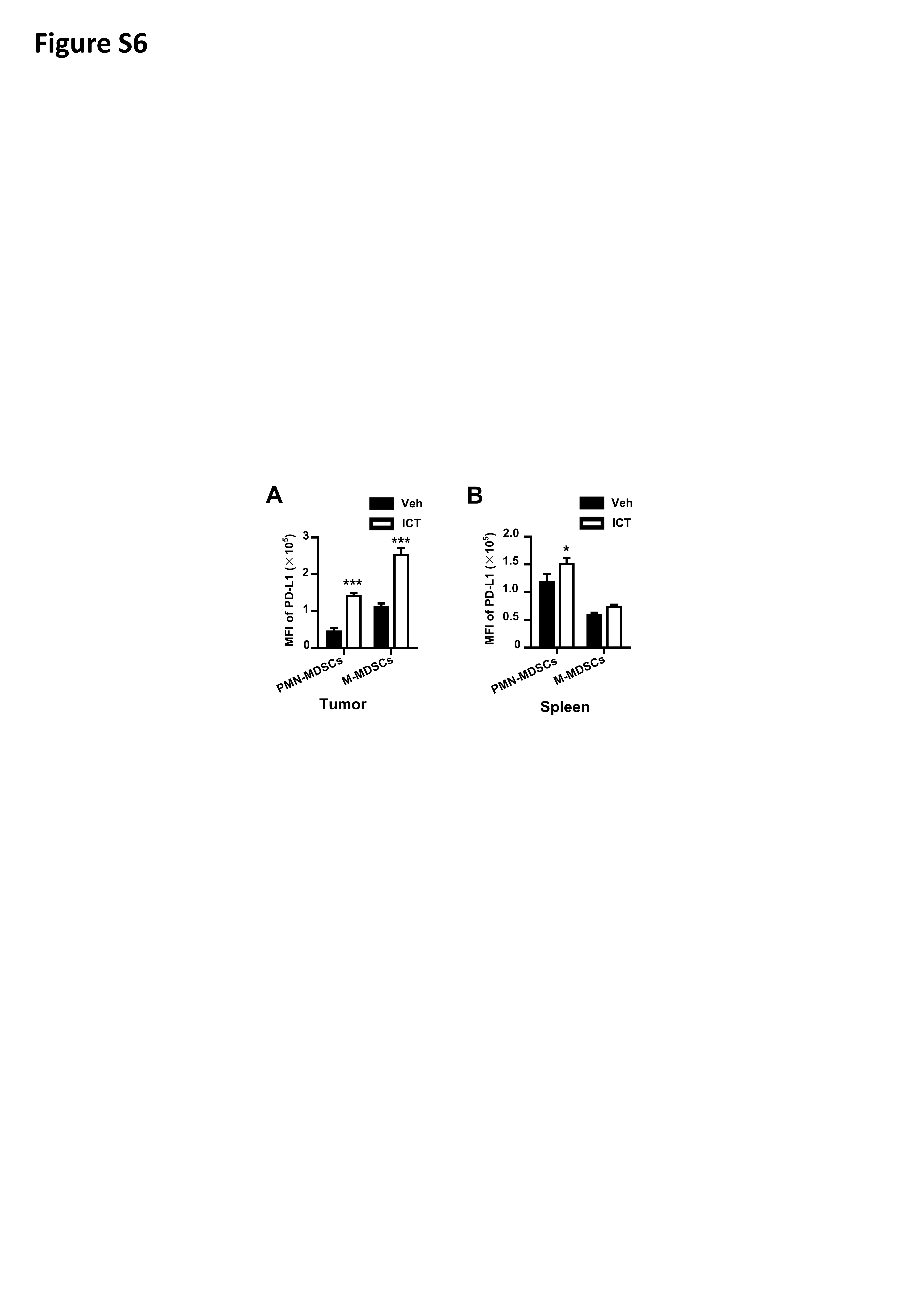

Supplement: Supplementary file 6 [file Image_6.tif]
