# Supplementary material for: Icaritin Induces Anti-tumor Immune Responses in Hepatocellular Carcinoma by Inhibiting Splenic Myeloid-Derived Suppressor Cell Generation
Source: Front Immunol. 2021 Feb 26;12:609295. doi: 10.3389/fimmu.2021.609295 (PMC7952329; doi:10.3389/fimmu.2021.609295)
Supplement: Supplementary file 7 [file Data_Sheet_1.pdf]

SUPPLEMENTERY MATERIALS AND METHODS

Apoptosis Assay

Hepa1-6 cells (1 × 10^5/well) were seeded in a 24-well plate in DMEM supplemented with 10% FBS, and allowed to attach overnight. The next day they were treated with icaritin or with DMSO (vehicle) for 48 h. Adherent and non-adherent cells were collected and resuspended in Annexin V-binding buffer. Cells were stained with Annexin V-FITC and DAPI (eBioscience) for flow cytometric measurement of apoptosis. Data were acquired using a Cytoflex S flow cytometer (Beckman Coulter) and analyzed using the FlowJo software (version 13; Tree Star).

Western Blot Analysis

Hepa1-6 cells (1 × 10^5/well) were seeded in a 24-well plate in DMEM supplemented with 10% FBS, and allowed to attach overnight. The next day they were treated with icaritin for 3 or 6 h. Cells were lysed using lysis buffer containing 0.1 mM PMSF and Protease Inhibitor Cocktail (Roche). Equal amounts of cellular protein were separated by 10% SDS-PAGE, and immunoblotted using the following antibodies: β-actin (AC-15; Boster Biological Technology), t-STAT3 (124H6; Cell Signaling Technology), p-STAT3 (D3A7; Cell Signaling Technology). Proteins were visualized using an ECL kit (Thermo Fisher Scientific).

Proliferation Assay

Hepa1-6 cells were seeded in 96-well plates (8 × 10^3 cells/well) in DMEM supplemented with 10% FBS and left overnight, prior to treatment with icaritin. Cell proliferation assays were performed using alamarBlue™ HS Cell Viability Reagent (Bestbio, China), according to the manufacturer's instructions. Absorbance was measured at 590 nm using infinite 200 PRO

(Tecan, Switzerland).

Flow Cytometry

Flow cytometry was performed as described in *Materials and Methods*. Details of the antibodies used for flow cytometry are listed in Supplemental Table 1. The phenotypes of tumor-infiltrating B cells, NK cells, macrophages and Tregs were evaluated by using B220, NK1.1, F4/80, CD4 and Foxp3.

Tumor Challenge and Treatments

1×10^6 Hepa1-6 were injected subcutaneously into the right flank of B6 mice. A total of five days after tumor cell transplantation, when tumors were palpable or subcutaneous tumors reached 100 mm^3, the mice were randomly divided into treatment groups. Icaritin (ICT; Beijing Shenogen Biomedical Ltd, China) treatment, was administered daily at 70 mg/kg by gavage. Corn oil was used as the vehicle control. For MDSC depletion with Gr-1 antibody, 9 days after tumor inoculation, mice were treated Anti-Gr-1 antibody (clone RB6-8C5; BioXCell) at 250 μg/mouse by intraperitoneal injection a total of three times at three-day intervals. Once tumors were palpable, tumor growth was monitored every other day for 17 days using calipers. Tumor volumes were calculated using the following formula: Volume = (length × $width^2$) / 2.

TABLE S1. Fluorochrome-Conjugated Antibodies Used in Flow Cytometry

Antigen	Specificity	Fluorochrome	Clone	Supplier
**CD45**	Mouse	Brilliant Violet 570	30-F11	Biolegend
**CD11b**	Mouse	AF700	M1/70	Biolegend
**Gr-1**	Mouse	FITC	RB6-8C5	Biolegend
**B220**	Mouse	Brilliant Violet 421	RA3-6B2	Biolegend
**Ly-6G**	Mouse	PE-CF 594	1A8	BD Horizan
**CD3**	Mouse	PE	17A2	Biolegend
**CD8a**	Mouse	eFluor 450	53-6.7	Invitrogen
**B220**	Mouse	Brilliant Violet 421	RA3-6B2	BioLegend
**IFN-γ**	Mouse	Alexa Fluor 488	XMG1.2	Invitrogen
**NK1.1**	Mouse	Alexa Fluor 700	PK136	BioLegend
**F4/80**	Mouse	APC	BM8	BioLegend
**CD274 (PD-L1)**	Mouse	PE-Cy7	10F.9G2	BioLegend
**Foxp3**	Mouse	APC	FJK-16s	Invitrogen
**CD4**	Mouse	PE-Cy7	GK1.5	BioLegend
**Lineage**	Mouse	PerCP-Cy5.5	145-2C11,	BD Pharmingen

**Cocktail**			M1/70, RA3-6B2,TER-11,RB6-8C5	
**Sca-1**	Mouse	Alexa Fluor 700	D7	Invitrogen
**CD117**	Mouse	PE-Cy7	2B8	BD Pharmingen
**Rat IgG2b κ**	Isotype ctrl	PE-Cy7	eB149/10H5	eBioscience
**Rat IgG1 κ**	Isotype ctrl	Alexa Fluor 488	eBRG1	Biolegend
**CD15**	Human	FITC	HI98	eBioscience
**CD14**	Human	Alexa Fluor700	M5E2	BD Pharmingen
**HLA-DR**	Human	PE-CF594	G46-6	BD Pharmingen
**CD115**	Human	APC	9-4D2-1E4	Biolegend

SUPPLEMENTARY FIGURE LEGEND

FIGURE S1 Body weight of orthotopic and subcutaneous Hepa mice. Body weights of orthotopic (**A**) and the subcutaneous (**B**) Hepa mice. Differences between groups were examined by Student's *t* test. Data were pooled from two experiments and n = 8 mice per group. Veh, Vehicle; ICT, Icaritin; Hepa, Hepa1-6 cells.

FIGURE S2 Icaritin has a negligible effect on apoptosis and proliferation of Hepa tumor cells. (**A**) Analysis of Hepa cell apoptosis. Numbers in the flow cytometric plots indicate the proportions of gated cell populations. (**B**) Western blot showing STAT3 protein expression in Hepa cells treated with icaritin. (**C**) Analysis of Hepa cell proliferation following icaritin treatment. Differences between groups were examined for statistical significance by two-way ANOVA, and corrected by Bonferroni's test. Data are representative of three experiments. Veh, Vehicle; ICT, Icaritin; p, phosphorylated; t, total; Hepa, Hepa1-6 cells.

FIGURE S3 Icaritin does not affect the frequencies of tumor-infiltrating B cells, NK cells, Treg cells or macrophages in Hepa mice._Gating strategies of tumor-infiltrating immune cells from orthotopic (**A**) and subcutaneous (**B**) Hepa mice. Frequencies of tumor-infiltrating B cells, NK cells, Mφ and Treg cells in $CD45^+$ cell populations from orthotopic (**C**) and subcutaneous (**D**) Hepa mice. Differences between groups were analyzed by two-way ANOVA, and corrected by Bonferroni's test. Data were pooled from two experiments and n = 8 mice per group. Veh, Vehicle; ICT, Icaritin; Hepa, Hepa1-6 cells; Mφ, macrophages.

FIGURE S4 Depletion of MDSCs with anti-Gr1 treatment abrogated the inhibitory effect on

tumor growth of icaritin. Images of subcutaneous tumors 26 days after inoculation (**A**). Mean tumor volume of subcutaneous Hepa tumor-bearing mice with and without anti-Gr-1 and icaritin treatment (**B**). Differences between groups were examined for statistical significance by two-way ANOVA, and corrected by Bonferroni's test. $^{*}P < 0.05$ and $^{**}P < 0.01$ compared with "Veh" group. Hepa, Hepa1-6 cells; ICT, icaritin; Veh, Vehicle.

FIGURE S5 The effect of icaritin on the accumulation of HSPCs in the bone marrow of Hepa mice. The numbers of bone marrow LSK and LK cells in the orthotopic (**A**) and subcutaneous (**B**) Hepa mice. Differences between groups were examined for statistical significance by Student's *t* test. Data were pooled from two experiments and n = 8 mice per group. HSPCs, hematopoietic stem and progenitor cells; Hepa, Hepa1-6 cells; ICT, icaritin; Veh, Vehicle; LSK, $Lin^{lo/-}Sca\text{-}1^{+}c\text{-}Kit^{hi}$; LK, $Lin^{lo/-}Sca\text{-}1^{-}c\text{-}Kit^{hi}$.

FIGURE S6 Icaritin increases the expression of PD-L1 by MDSCs in Hepa mice. PD-L1 MFI of tumor (**A**) and splenic (**B**) MDSCs from subcutaneous Hepa mice Differences between groups were examined for statistical significance two-way ANOVA corrected by Bonferroni's test. $^{*}P<0.05$ and $^{***}P<0.0001$. Data were pooled from two experiments and n = 8 mice per group. Veh, Vehicle; ICT, Icaritin; MFI, mean fluorescence intensity; M-MDSCs, mononuclear myeloid-derived suppressor cells; PMN-MDSCs, polymorphonuclear myeloid-derived suppressor ce
